# Realization path and connotation of the Healthy China strategy: macroscopic perspective of dietary structure and the entry of individual health consciousness

**DOI:** 10.1186/s12889-024-18557-z

**Published:** 2024-04-23

**Authors:** Xiaohua Zhu, Yan Zhang, Yuanyuan Zhu, Youhua Guo, Yunjin Zhang, Bin Wen

**Affiliations:** 1 Institute of Geographic Sciences and Natural Resources Research, Chinese Academy of Sciences, 100101 Beijing, China; 2 Key Laboratory of Geographic Process Analysis & Simulation Hubei province, Central China Normal University, 430079 Wuhan, China; 3 Department of Rehabilitation of General Hospital of Guangdong Provincial Hospital of Chinese Medicine, Guangdong Provincial Hospital of Chinese Medicine, 510120 Guangzhou, China; 4 Beijing Freedo Technology Co., Ltd, 100080 Beijing, China; 5 Sinosoft Co., Ltd, 100190 Beijing, China

**Keywords:** Dietary structure, Health consciousness, Realization path, Health China

## Abstract

**Background:**

Dietary rationality and health concept have certain influence on individual health level. This study aims to explore the characteristics and existing problems of Chinese residents’ health behaviors from both macro and micro perspectives, and explore the feasibility and realization path of Healthy China strategy.

**Methods:**

We utilized regression models to evaluate the correlation between diet and the risk of disease causes of death. By use of the linear regression analysis model, we distinguished the impact of each dimension on health literacy index at the individual level. Then, we explored the influential factors of the diet health index using the binary logit regression model.

**Results:**

Increased consumption of animal-derived foods in China has contributed to the burden of non-communicable diseases. The individuals’ health awareness is still weak, and the health literacy index is greatly affected by the diet, while the individual gender and age are positively correlated with the diet health index, and the individual body mass index (BMI) level is negatively correlated with the diet health index.

**Conclusions:**

This study provided a comprehensive understanding of existing problems of Chinese residents’ health behaviors. We have proposed a path model for the implementation of the Healthy China strategy from the perspectives of “diet health, physical health, conceptual health and environmental health,” which is also a great contribution to the world.

**Supplementary Information:**

The online version contains supplementary material available at 10.1186/s12889-024-18557-z.

## Introduction

Health is a crucial indicator of national development level and social stability [1]. Over the past century, remarkable progress has been made in global health [2]. However, significant variations in health levels persist among countries and regions [3]. Furthermore, as the current global health systems grapple with ensuring seamless provision of healthcare services [4], the role and responsibility of individuals in promoting global health is increasingly crucial [5, 6]. Therefore, improving individual health literacy, particularly in densely populated regions, will be a key driver in building a global community of life.

Health is affected by numerous factors such as medical, income, and education [7]. On one hand, various research perspectives and methods have been extensively employed to analyze disparities in individual health awareness levels [8–10]. Most of them have demonstrated that environmental and cognitive differences are the primary factors impacting health levels [11–14]. On the other hand, increasing attention has been given to the impact of individual behaviors on health, encompassing daily habits such as diet and exercise [15–16]. Substantial evidence suggests that adopting a healthy and diverse dietary pattern can decrease the risk of major diet-related chronic diseases, such as cardiovascular and cerebrovascular diseases [17]. Shifting to a Mediterranean diet versus a Western diet led to significantly different disease burdens [18, 19]. Furthermore, the role of health strategies in achieving Universal Health Coverage (UHC) has been recognized [20–21]. The United States has been in the stage of promoting individual health literacy to advance universal health [22].

**Fig. 1:**
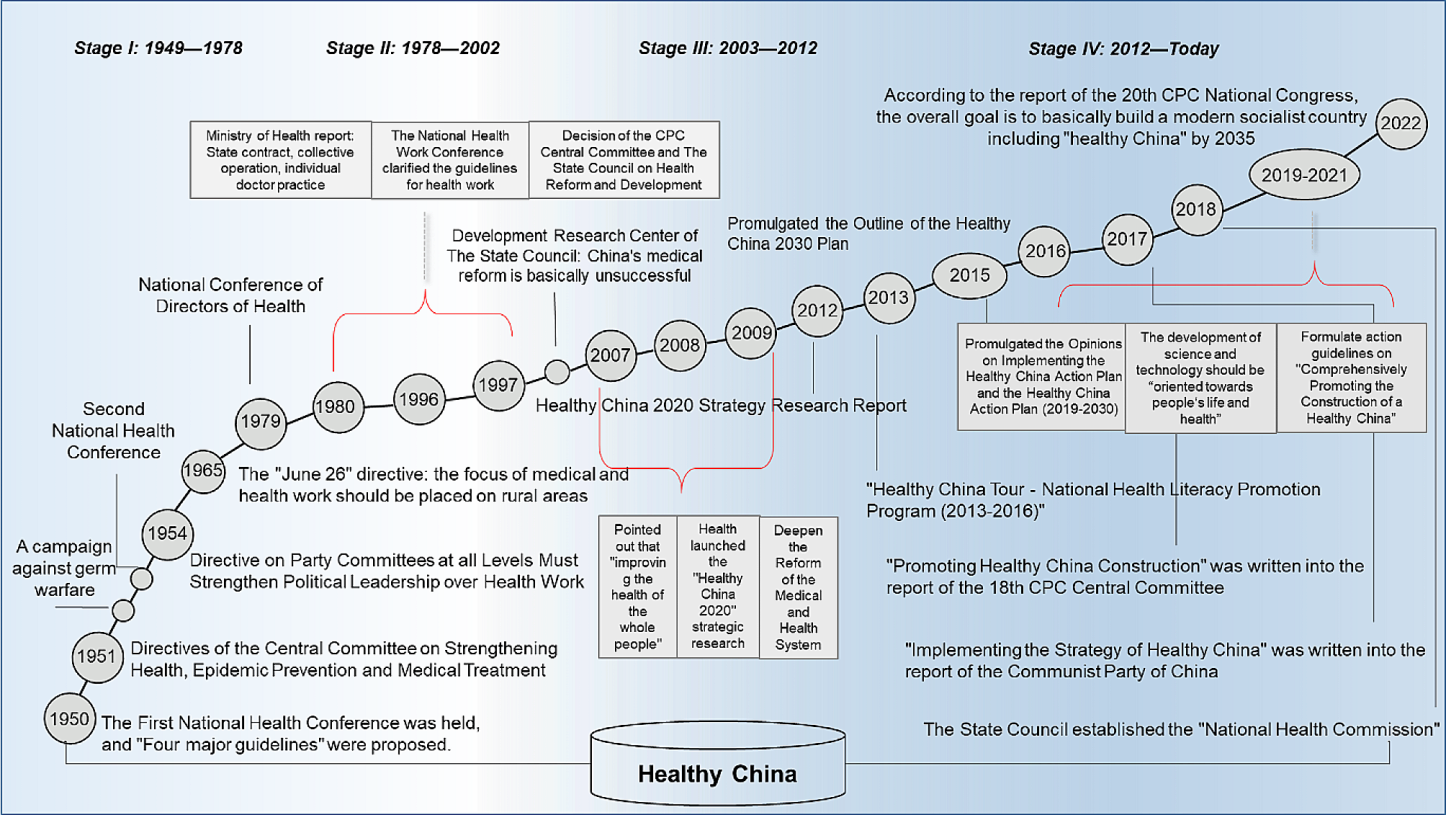
Development history of Healthy China (1949-present). *Note* The data used in Fig. 1 was obtained from National Health Commission of the People’s Republic of China, http://www.nhc.gov.cn/

China accounts for one-fifth of the global population. The life expectancy of the Chinese has gradually risen in the past 10 years. However, problems of malnutrition and excess still exist, and the prevalence of chronic diseases continues to increase [23]. China’s health strategy has evolved over 70 years; the Healthy China action has shown initial positive outcomes (Fig. 1). Nevertheless, a strategy centered around “curing diseases” falls short in meeting the healthcare demands of the public, health level inequality still exists [24], warranting a reform in health policy that aligns with the current public health needs in China.

There is mutual influence and restriction between diet, consciousness and health. Previous studies on individual health have primarily concentrated on analyzing the health level of a certain group of people and its influencing factors [25, 26]. However, these studies failed to comprehensively examine health behavior of different groups of people, including the relationship between various factors and health levels, particularly the interplay between dietary habits and individual health consciousness, as well as the problems existing in public health behavior and awareness. Additionally, insufficient attention has been given to the conflict and equilibrium between the current national health policy and individual health consciousness and behavior.

To address this gap, from the multi-dimensional perspective of “diet-consciousness-health-life community”, this study explored the macro health effects of changes in the Chinese diet structure, and combined individual health behavior awareness from a micro perspective to analyze the trends and issues in individual health behavior and awareness. The Healthy China Strategy is a strategy to realize public health from the perspective of the Chinese public, and represents a comprehensive approach towards ensuring lifelong health for all individuals in China from the micro perspective. The contributions of this study are as follows: (1) We conducted the first survey on the health awareness and behavior of Chinese public. (2) We analyzed the characteristics and existing problems of health behaviors among Chinese from both macro and micro perspectives; (3) Micro implementation is the cornerstone of macro policy implementation. Based on analysis of macro and micro perspective, we put forward the more scientific and feasible approach in promoting Healthy China action from different perspectives.

## Methods

### Study design and sampling

First, a national, cross-sectional, representative survey with a random stratified sampling design was conducted involving Chinese residents aged 12 years and older. The participants come from different provinces, districts, counties and villages in China, including diverse individuals in terms of age, education level, and occupation. The samples are stratified according to different ages and education levels, and geographical location information is added to ensure representative sampling from diverse regions and populations. The micro-level health behavior analysis is an important way to reflect Chinese residents’ health behavior issues and health literacy level.

Second, macro analysis can observe the dynamic evolution of the dietary structure of the Chinese public, identifying prevalent issues in the Chinese diet, and to carry out to individual lifestyle issues through micro-level behavior analysis. We collected the macro-scale diet data primarily from the China Statistical Yearbook, China Rural Statistical Yearbook and China Social Statistical Yearbook spanning 1987 to 2020. We referred the GBD 2017 Diet Collaborators (2019) [27] to identify dietary risk factors influencing mortality from the leading 10 diseases in China. Food items were categorized into eight types, namely grains, vegetables, fruits, oilseeds, sugars, eggs, milk, and meat. The health data primarily originated from the China Statistical Yearbook and China Health Statistical Yearbook spanning 1987 to 2020. The data types mainly related to the composition of the ten major diseases that causing death in China. We performed correlation analysis to examine the relationship between food consumption and the death rate caused by ten diseases among the Chinese. Additionally, linear regression analysis was carried out using the food items identified as significant predictors to investigate their association with the top four diseases (Formula 1). The standardized regression coefficient obtained from the linear regression analysis was used to quantify the impact of highly correlated food consumption on disease-specific mortality.

1 $${y_i}={\beta _0}+{\beta _1}{x_i}+{\mu _i}$$

where, ${y_i}$is the mortality rate of 4 diseases with strong correlation, and ${x_i}$ are the food types with high correlation with disease mortality, namely, grain, fruits, oilseeds, eggs, milk and meat. ${\beta _0}$ is the intercept of the model, and ${\beta _1}$ is a set of regression coefficients corresponding to the respective variables, ${\mu _i}$ represents the error term.

### Study population

The research team obtained questionnaire data on individual health awareness and behavior through an online survey distributed on social media platforms from July 2022 to February 2023. A total of 1719 questionnaires were collected. The wording of the material was provided only in Chinese and was not specifically targeted to low-literate individuals. Groups of different educational levels and different age groups were considered to increase representation.

### Measures

The questionnaire focuses on understanding the general public’s awareness and health-related behavior from the perspective of daily life. A total of 70 survey questions covering seven aspects were designed, including individual health awareness and behavior pertaining to work and rest; daily diet; tobacco, alcohol, nutrition; excretion (stool and urine); food quality; exercise, physical and mental conditions; and health cognition (Table S1).

#### Health literacy index

We referenced the monitoring and evaluation standards of Chinese health literacy and the “Chinese Citizens’ Health Literacy - Basic Knowledge and Skills (2015 edition)” as the basis [28], and assigned the seven indicators to 1–5 points (Table S1). The questionnaire was designed with reference to Chinese residents’ health literacy monitoring and HLY-EU-Q16 [29, 30]. We evaluated the level of individual health literacy by using 70 indicators.

The questionnaire provides a maximum of five options for participants to choose from for each indicator. Each option under a given indicator was assigned a maximum score of five points. The overall health literacy score of an individual was obtained by summing the scores of all indicators. This study compared the individual’s health literacy score with the total score of the evaluation index system used to assess Chinese health literacy levels. The quotient resulting from this comparison represents the individual’s health literacy index. Additionally, we compared the scores of the seven dimensions of health awareness and behavior with the standard total score. We obtained distinct indices for each aspect: the health work and rest index, diet health index, health care index, health excretion (stool and urine) index, food health concern index, health exercise index, and health cognition index.

#### Demographic and socioeconomic characteristics

The gender structure of the respondents was 44.04% male (757) and 55.96% female (962). Respondents’ ages ranged from 12 to 83 years, with an average age of 50.71 years. Educational levels were categorized based on the highest completed level of education one year prior to survey completion. Basic school, primary educations, lower secondary, upper secondary, and vocational educations reflect the first and second education levels. Medium length educations including short and medium length tertiary, and bachelor’s educations, and higher length education containing master’s level and PhD-level educations reflect the third and fourth levels of education. We converted the education years of the samples according to the education years of different stages in China. The average education time of the respondents is 17.0, which is between the bachelor degree and the master degree.

#### Health awareness and behavior

The survey included various measures of health behavior such as work and rest conditions, daily diet, smoking habits, alcohol consumption, and physical activity. Work and rest conditions were assessed based on factors such as rest time and sleep status. Daily diet was evaluated with respect to meal patterns and food choices. Tobacco, alcohol, and nutritional products were the criteria of whether the individual smoked and drank alcohol and took nutritional health products. The condition of defecation is concerned about the individual’s physical condition and the degree of cognition of defecation. Food quality primarily examined individuals’ trust in the quality of the food they consumed. Exercise and physical and mental condition aimed to investigate the intensity and time of individual exercise, and to evaluate the physical and mental condition of individuals through fatigue and working hours. Health cognition was assessed based on the individual’s cognition of medical treatment, medical hygiene and individual health level. Self-reported height and weight data were obtained to allow calculation of body mass index (BMI, BMI = weight (kg) divided by the square of height (m)). The classification of BMI mainly refers to the standard defined by the Health Commission of the People’s Republic of China (BMI < 18.5: underweight; 18.5 ≤ BMI < 24.0: normal; 24 ≤ BMI < 28: overweight; BMI ≥ 28: obesity).

### Statistical analysis

The reliability coefficient of the questionnaire was 0.745, and the KMO value of the validity test was 0.843, indicating that the design of questionnaire questions and the collected sample results are reliable. We categorized the sample health literacy index as either above or below the average level, and labeled the two groups as “good” and “poor”.

With the health literacy index of the survey samples as the dependent variable and the score index of various indicators as the independent variable, linear regression analysis was conducted (Formula 2). The standardized regression coefficient in the linear regression analysis results was used to quantify the evaluation of indicators strongly correlated with the individual health literacy index. Binary Logistic regression, a suitable method for exploring the relationship between categorical variables, was employed to analyze the relationship between different demographic characteristics and diet health index (Formula 3). When the diet health index is higher than the average, *Y* = 1; when the diet health index is lower than the average, *Y* = 0. We adopt demographic characteristic variables to make adjustments, such as: age, gender, degree, BMI, the reason for choosing these variables is that previous studies found that these variables are related to health status. Additionally, we conducted robustness analysis using the binary Probit model for the same dependent and independent variables, and the results were consistent with those of the Logit model (Table S2). All analyses were performed using Stata 17.0 and SPSS 22.0. *p* < 0.05 was considered significant, and p values were both bilateral.

2 $${Y_i}={\beta _0}+{\beta _1}{X_i}+{\mu _i}$$

where, ${Y_i}$ is the dependent variable, that is, the health literacy index; ${X_i}$ is the respective variable, that is, the score index of 7 aspects in the questionnaire; ${\beta _0}$ is the intercept of the model, and ${\beta _1}$ is a set of regression coefficients corresponding to the respective variables, ${\mu _i}$ represents the error term.

3 $$Logit({P_j})=\ln \left[ {\frac{{{p_j}}}{{1 - {p_j}}}} \right]={\beta _0}+{\beta _1}{X_j}+{\mu _j}$$

where, ${p_j}$ refers to the probability that the sample’s diet health index is at a certain level, ${\beta _0}$ is the intercept of the model, and ${\beta _1}$ is a set of regression coefficients corresponding to their respective variables. Among them, the independent variables${X_j}$ defined in this study are demographic characteristic variables: age, gender, degree, Body Mass Index, ${\mu _j}$ represents the error term.

## Results

### Chinese dietary structure has entered the stage of degenerative diseases

**Fig. 2:**
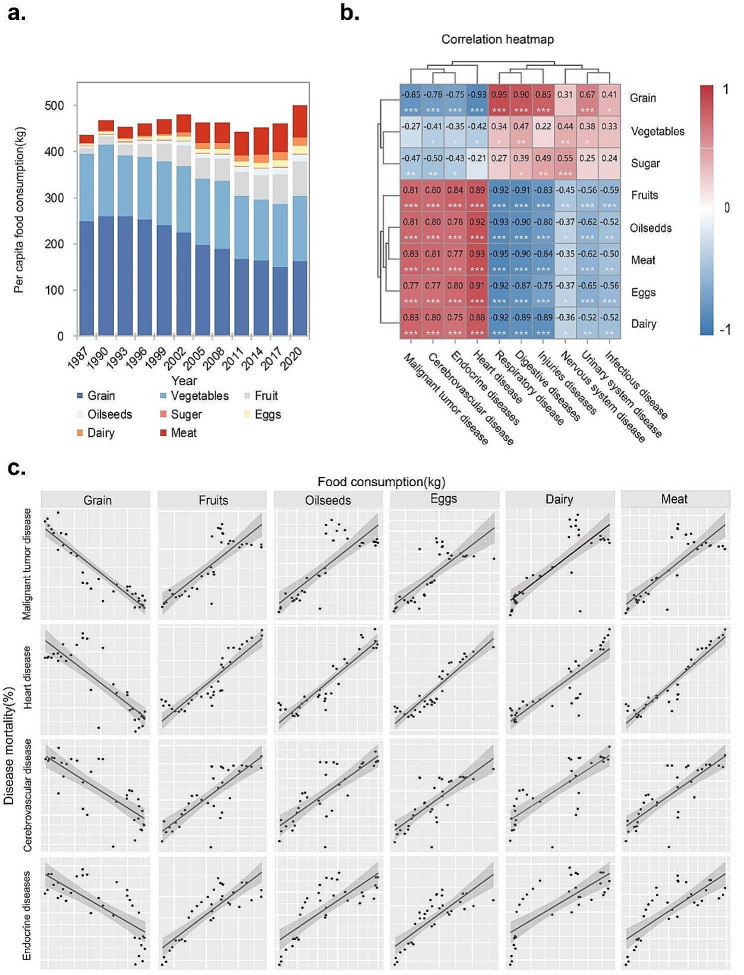
Changes of dietary structure and its health effects in China from 1987 to 2020. **a**: evolution of Chinese dietary structure from 1987–2020; **b**. the correlation between the consumption of various types of food and the rate of ten major diseases; **c**. linear regression of food consumption and disease rate

From 1987 to 2020, Chinese dietary structure has changed from plant-based diet to animal-based diet (Fig. 2a). Malignant tumors, heart disease, cerebrovascular disease, endocrine and nutritional metabolic diseases were significantly positively associated with fruit consumption, animal-derived foods (eggs, dairy, meat), and oilseeds, while significantly negatively associated with grain consumption (Fig. 2b). With every 1 kg increase in per capita fruit consumption, the proportion of deaths due to malignant tumors, heart disease, cerebrovascular disease, and endocrine and nutritional metabolic diseases will increase by 0.14%, 0.2%, 0.07%, and 0.03%, respectively. For oilseeds, the mortality rate due to malignant tumors, heart disease, cerebrovascular disease, and endocrine and nutritional metabolic diseases increased by 0.76%, 1.18%, 0.38%, and 0.15%, respectively. For egg, dairy and meat, malignant tumors, heart disease, cerebrovascular disease, endocrine and nutritional metabolic diseases accounted for 0.62%, 0.38% and 0.15% of the total number of deaths, 0.99%, 0.55% and 0.23%, 0.31%, 0.19% and 0.07%, 0.13%, 0.07% and 0.03%, respectively. For grain, the mortality rate due to malignant tumors, heart disease, cerebrovascular disease, endocrine disease, and nutritional metabolic disease will decrease by 0.06%, 0.09%, 0.03%, and 0.01%, respectively (Fig. 2c). The Chinese have entered a critical period characterized by the prevalence of nutrition-related chronic diseases caused by changes in dietary structure, emphasizing the urgent need for dietary structure improvement and intervention.

### Analysis of individual health literacy characteristics in China

#### Overall feature analysis

**Fig. 3:**
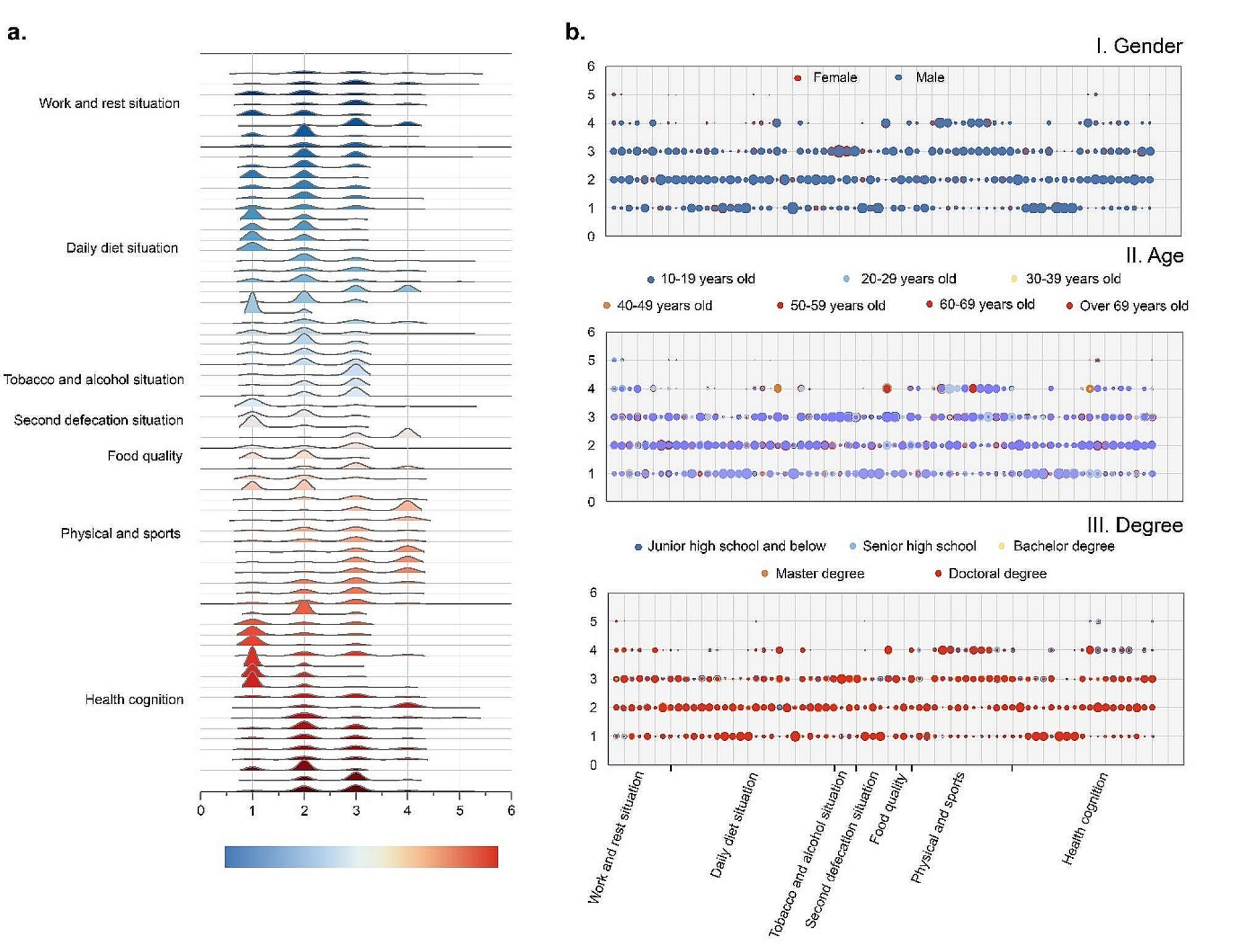
Analysis of individual health literacy. **a**: overall distribution of each indicator option; **b**: option distribution of each index under demographic characteristics

The overall average level of health awareness and behavior in the survey samples was 0.71, but there were in the different indicators (Fig. 3a). In terms of work and rest, the proportion of samples in the “go to bed before twelve o ‘clock,” “often brush the mobile phone before going to bed, “occasionally stay up late, insomnia” and “sleep duration of about 6–8 hours” are in the forefront, which are higher than 40%, except for the proportion of samples in the “get up before 7 o ‘clock every morning” is 30–40%. The proportion of the total sample with a “sleep duration of about 6–8 hours” reached 73.2%. The proportion of “have the habit of taking lunch breaks all the year round” and “occasionally insomnia” samples were the highest, reaching 43.4% and 39.7%, respectively. In terms of daily diet, most survey samples reported regular eating patterns with relatively large dinners, occasional eating out, and usual drinking water. Food choices are typically evenly split between meat and vegetables, with frequent fruit consumption, preference for strong flavors, and occasional consumption of processed packaged foods. In terms of diet awareness, the proportion of samples who eat until they feel full and understand the harm of some “three whites” is higher, except for the proportion of “often eat fruit” in 40%, the remaining proportion is higher than 50%. The proportion of people who abstain from consuming late-night snacks is high.

In terms of tobacco and alcohol, nutrition and health products, defecation and food quality, the sample characteristics showed that “no smoking,” “no drinking,” “no health products,” “smooth defecation every day,” “occasionally get hemorrhoids” and the proportion of general trust in food quality was higher, reaching over 50%. Regarding exercise, the prevalence of behaviors such as “frequent long periods of sitting,” “limited sun exposure,” “occasional short-duration exercise,” and the belief that a healthy body does not require weight loss ranked highest, exceeding 40%. In terms of physical and mental conditions, the majority reported average levels of life stress, anxiety, work intensity, and fatigue, with some experiencing occasional symptoms, but no instances of depression or tinnitus. The majority agree that overnutrition can harm health, chronic patients need lifelong medication, surgical success does not guarantee successful treatment, and normal indicators do not necessarily indicate good physical health. In terms of trust in doctors and hospitals, as well as the understanding of adverse drug reactions and excessive medical treatment, most are mediocre. Regarding medical treatment, the proportion of samples of “tell the doctor all when seeing a doctor,” “seek medical treatment in time”, “check-up once a year”, “integration of traditional Chinese and Western medicine” and “hospital is auxiliary, oneself is the main” was higher. The cognitive degree of rehabilitation treatment and misdiagnosis rate was “know a little.” Most of the samples do not believe that “science and technology can cure any disease”, and most of them think that they are sub-healthy.

#### Category analysis of individual characteristics

(1) Gender.

The health awareness and behavior of men and women exhibit consistent tendencies in various aspects (Fig. 3b). In terms of work and rest, male and female samples showed similar characteristics to the overall sample. Regarding sleep quality, 40.4% of women consider their sleep quality to be average, while 38.6% of men perceive theirs as good. In regards to daily diet, both men and women exhibited a pattern similar to that of the overall sample. Concerning the negative effects of gutter oil, men showed a relatively higher proportion, while women’s proportion was generally understood. As for fruit, women eat it often, while men eat it occasionally. When it comes to late night snacks and colored or carbonated drinks, men report consuming them “occasionally,” while women tend to report consuming them “almost never.” In regards to dinner richness, most men described it as rich, whereas women described it as average.

Regarding alcohol consumption, 52% of men reported drinking occasionally, whereas 75.6% of women reported abstaining from alcohol. Regarding the frequency of sun exposure, 59% of women claimed to be “not active at all,” whereas 51% of men claimed to be “often active.” In regards to other conditions, male and female characteristics also exhibited homogeneity with the overall population. Regarding working hours, 50% of women work less than eight hours, while 42% of men work eight to ten hours. In regards to health cognition, women tended to believe that “the success of surgery and the cure of the disease are two different things,” whereas men tended to believe that “the success of surgery is a partial cure of the disease.”

(2)Age.

The survey group’s age was categorized into 10-year intervals, revealing significant variations in individual health awareness and behavior (Fig. 3b). In terms of work and rest, as age increased, the samples indicated that individuals went to bed and woke up earlier, had longer naps, and experienced poorer sleep quality. Additionally, all age groups displayed a tendency to align more closely with the overall sample’s dietary characteristics. Dietary preferences tend to become lighter with increasing age, and individuals over the age of 69 generally abstain from consuming carbonated beverages.

In terms of tobacco, alcohol and defecation, the proportion of people over 69 years of age who regularly took health products was significantly higher than the other samples. As age increases, concern regarding food quality gradually rises. With regards to physical activity, individuals in the 20–29 age group had a higher proportion of prolonged sitting. With the increase in age, the sun exposure and exercise are also changed to “often sun” and “regular exercise”. Regarding physical and mental well-being, individuals aged 20 to 39 perceived higher levels of life pressure. In terms of health cognition, with the increase of age, the responsibility of individual health is supplemented by the hospital, and the main is transformed into “relying on the hospital”, the degree of trust in the hospital is decreasing, and the cognition and attention degree of health such as “adverse drug reaction,” “misdiagnosis rate,” and “physical examination” are gradually increasing. The proportion of samples in the 20–59 age group who said they were “sub-healthy” was higher, while the rest of the group considered themselves “very healthy” or “relatively healthy.”

(3)Degree.

The education levels of the survey group were categorized into five stages: junior high school or below, senior high school, undergraduate, master’s, and doctorate degrees. The level of health literacy increased gradually with higher education levels, leading to significant differences in individual health awareness and behavior (Fig. 3b). Regarding work and rest, as the educational level increases, there is a trend of going to bed and waking up later, and having longer lunch breaks. With higher education levels, individuals pay more attention to the rationality of their diet, and more people understand the risks of adulterated cooking oil and consume fruit regularly. In terms of dietary awareness, the samples exhibited similar characteristics to the overall sample.

In terms of tobacco and alcohol, nutrition and health products, defecation and food quality, the sample characteristics of all education levels mostly exhibited the characteristics to the overall sample. In terms of exercise, with the rise of educational levels, sedentary and sun exposure has also changed to “basically sedentary every day” and “basically not actively sun-bathed.” Regarding physical and mental conditions, higher education levels are associated with increased life pressure and longer, more intense work hours. With the rise of educational level, the individuals’ cognition of health and medical treatment, such as “cognition degree of rehabilitation medical treatment,” “adverse drug reactions,” “misdiagnosis rate,” has gradually increased.

### Analysis of influencing factors of individual health awareness and behavior

#### Characteristics of individual health literacy index of the survey sample

**Fig. 4:**
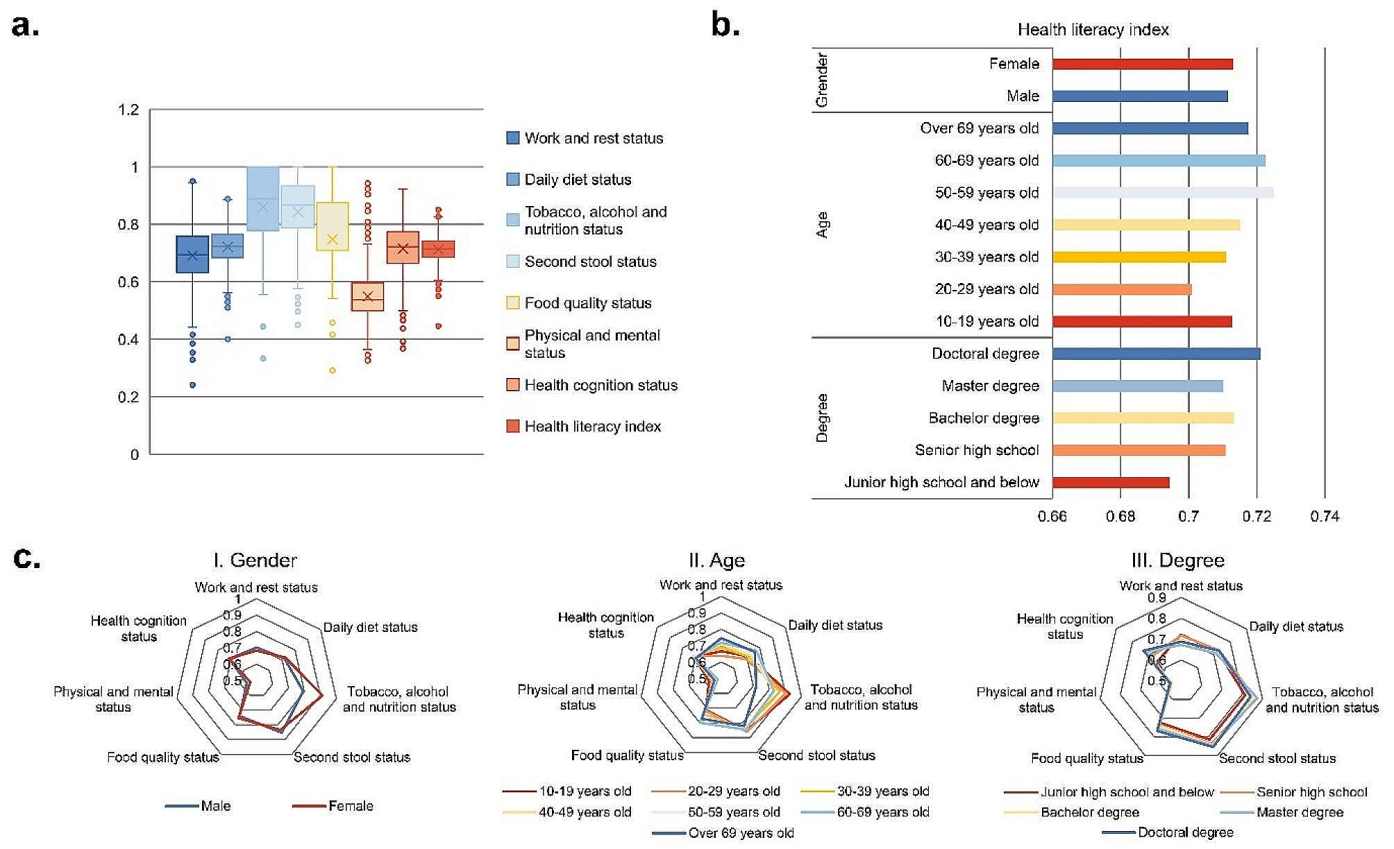
Individual health literacy index. **a**: overall sample scores in each dimension; **b**: health literacy score under demographic characteristics; **c**: the score of each dimension under each demographic characteristic

The average level of individual health literacy index in the survey samples was 0.7156, however, the sample’s health literacy index varied across different demographic characteristics (Fig. 4a, b). In general, 891 individuals (51.8% of the total sample) scored above the average personal health literacy index, with the overall health literacy level of the sample falling between 0.45 and 0.85. The average individual health literacy index for males and females in the survey sample was 0.7116 and 0.7129, respectively. Regarding education level, the survey samples indicated that the individual health literacy index gradually improved with higher levels of education. Regarding age, the survey sample population showed that the health literacy index of individuals gradually improved with increasing age.

Significant differences were observed in the scores of all aspects of the individual health behavior and awareness questionnaire among the survey samples (Fig. 4a, c). Among the seven aspects of health literacy scores in the survey sample, the average index for tobacco, alcohol, nutrition, and stool was higher, exceeding 0.8. However, the average index for physical and mental conditions was the lowest at 0.55, indicating a weak inclination towards regular physical exercise among the residents. The index characteristics of each gender, degree and age are similar to the characteristics of the total sample, showing that the average index of tobacco, alcohol, nutrition and stool is higher, and the average index of exercise and physical and mental conditions is lowest. In terms of work and rest, with the increase of degree and age, the average index showed a trend of decrease and increase, respectively. Regarding health cognition, there was an increasing trend in the average index with improving degree; however, it did not increase with advancing age. In terms of gender, there was no significant difference between men and women in the average index of all indicators. However, the average index for men was significantly lower than that for women in tobacco, alcohol, and nutrition, which may be attributed to the presence of smoking and drinking habits among males.

#### Analysis of influencing factors of individual health literacy index of the survey sample

**Fig. 5:**
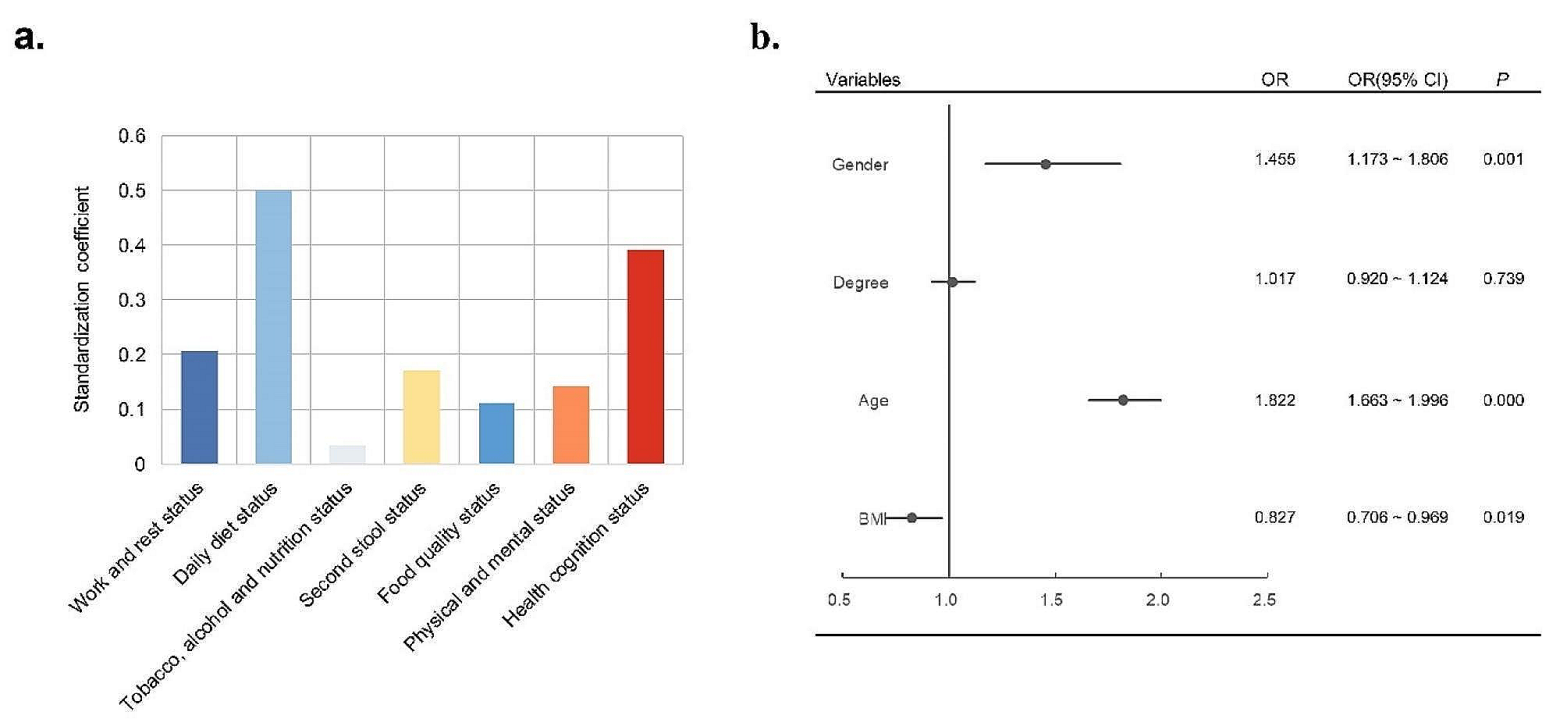
Individual health literacy index linear regression and individual diet health index binary Logit regression. **a**: correlation between each dimension and health literacy index; **b**: logit regression of each variable and diet health index

The individual health literacy index of the survey samples was affected by varying degrees by different aspects of health behaviors and awareness (Fig. 5a). The score index of all aspects was positively correlated with the individual health literacy index. For the individual health literacy index and the seven aspects of the survey samples, the regression standardization coefficients were 0.207, 0.502, 0.034, 0.170, 0.113, 0.142, and 0.392, respectively. However, the linear regression results of R showed that only the daily diet (0.500) and health cognition (0.544) of individuals had significant associations with the health literacy index. Among them, the daily diet exhibited a high regression standardization coefficient, suggesting that it is the primary determinant of the individual health literacy index within the survey sample.

Gender, degree, age, and BMI were taken as independent variables, while diet health index was taken as dependent variable for binary Logit regression analysis (Fig. 5b). The Table S3 show that gender, degree, age, and BMI can explain the differences in diet health index. In the final analysis, the regression coefficient for gender was 0.375, indicating women tend to have higher index. The regression coefficient value of degree was 0.017, but it did not show significance (z = 0.333, *p* = 0.739 > 0.05), suggesting that degree is not significantly correlated with the diet health index. The regression coefficient of age was 0.600, indicating a significant positive correlation between age and the diet health index. The change (increase) in diet health index was 1.822-fold with a one-unit increase in age. The regression coefficient for BMI was − 0.190, indicating that a significant negative correlation between BMI and the diet health index. With each one-unit increase in BMI, there was a 0.827 times decrease in the diet health index.

## Discussion

Health is an important guarantee for human survival and sustainable development. This study explored the health effects of the dietary structural evolution from 1987 to 2020, and analyzed the level of individual health literacy index and the influencing factors of dietary health index based on 1719 samples.

Recent studies have gradually focused on the correlation between diet and health. The global diet has shifted towards a wealthier dietary pattern [31]. Research indicates that the global risk of disease resulting from inappropriate diets surpasses that of any other factor, including smoking [27]. This study identified the dynamic evolutionary characteristics and issues at the macro level, observing a gradual shift towards a Western-style (affluent) dietary pattern in China. Despite the continuous efforts of the Chinese government to improve the inadequate healthcare system and the increasing healthcare expenditure, the number of patients in China, especially the proportion of non-communicable diseases, continues to rise [32]. Studies have found that the risk of cardiovascular disease in Chinese is closely related to dietary factors [33]. Therefore, adjusting the diet to a nutritious and healthy model will be a priority for achieving public health.

The individual consciousness are important factors affecting the residents’ physical health. This study implemented health behaviors and awareness among individuals at the micro level, and found that public attention to health awareness and behavior in China is still inadequate, with the average individual physical activity index reaching only 0.55. Furthermore, we determined that the diet health index has the greatest impact on the individual health literacy, and a significant negative correlation exists between individual BMI and dietary health index. BMI is significantly linked to the risk of cardiovascular disease [34]. Currently, there are 600 million obese and overweight people in China [35]. The unreasonable diet, the weak health awareness, and the continuous rise of BMI are constantly impacting the reform of China’s medical and health system. Therefore, it highlights the urgency and importance of changing public health awareness to realize a Healthy China strategy.

The evolution of the Chinese diet has deviated from the environmentally friendly model. Some studies have showed that if current consumption continues, China’s existing cultivated land resources will be insufficient to guarantee the sustainable consumption of 1.4 billion Chinese [36, 37]. Moreover, global adoption of the Western diet would lead to the dual crisis of environmental unsustainability and increased disease risk [38]. The Lancet diet could play a crucial role in achieving the global Sustainable Development Goals in the Anthropocene era [39]. The current dietary behaviors and health awareness in China still diverge significantly from a healthy diet [40]. Therefore, urgent attention must be given to the practical feasibility of health awareness adjustments that promote public health.

This study has several limitations. First, it used average annual food consumption data for China, the food consumption of detailed groups is not divided and the sources of dietary intake are not accurately assessed. Second, the sample data is limited. The development of the national policy implementation pathway needs to consider individuals with diverse characteristics in China. In this study, the average educational level of the sample exceeds the national average. Additionally, the average age of the sample (50.71) surpasses that of the entire Chinese population (38.8). Moreover, there is a certain deviation from the sex ratio in the total sample. However, it’s important to highlight that the sample encompasses individuals with diverse characteristics, including age, education, occupation, and gender, indicating that the research findings hold certain reference significance for the implementation of the Healthy China action. Furthermore, a comprehensive survey report covering the health behaviors of Chinese public in revealed issues such as low health literacy, a small proportion of individuals engaging in regular physical exercise, unhealthy dietary patterns, and weak health awareness, which align with our findings (https://tuhi.tsinghua.edu.cn/en/index.html). Third, this study is based on cross-sectional survey data, which limits its ability to capture long-term changes. Finally, it should be noted that the connection of macro and micro-level analyses is the combination of two different perspectives, the statistical analyses done in this study couldn’t completely inform the “causation” from micro-level behavioral change towards macro-level changes, which should be further studied in the future.

## Public health implications

**Fig. 6:**
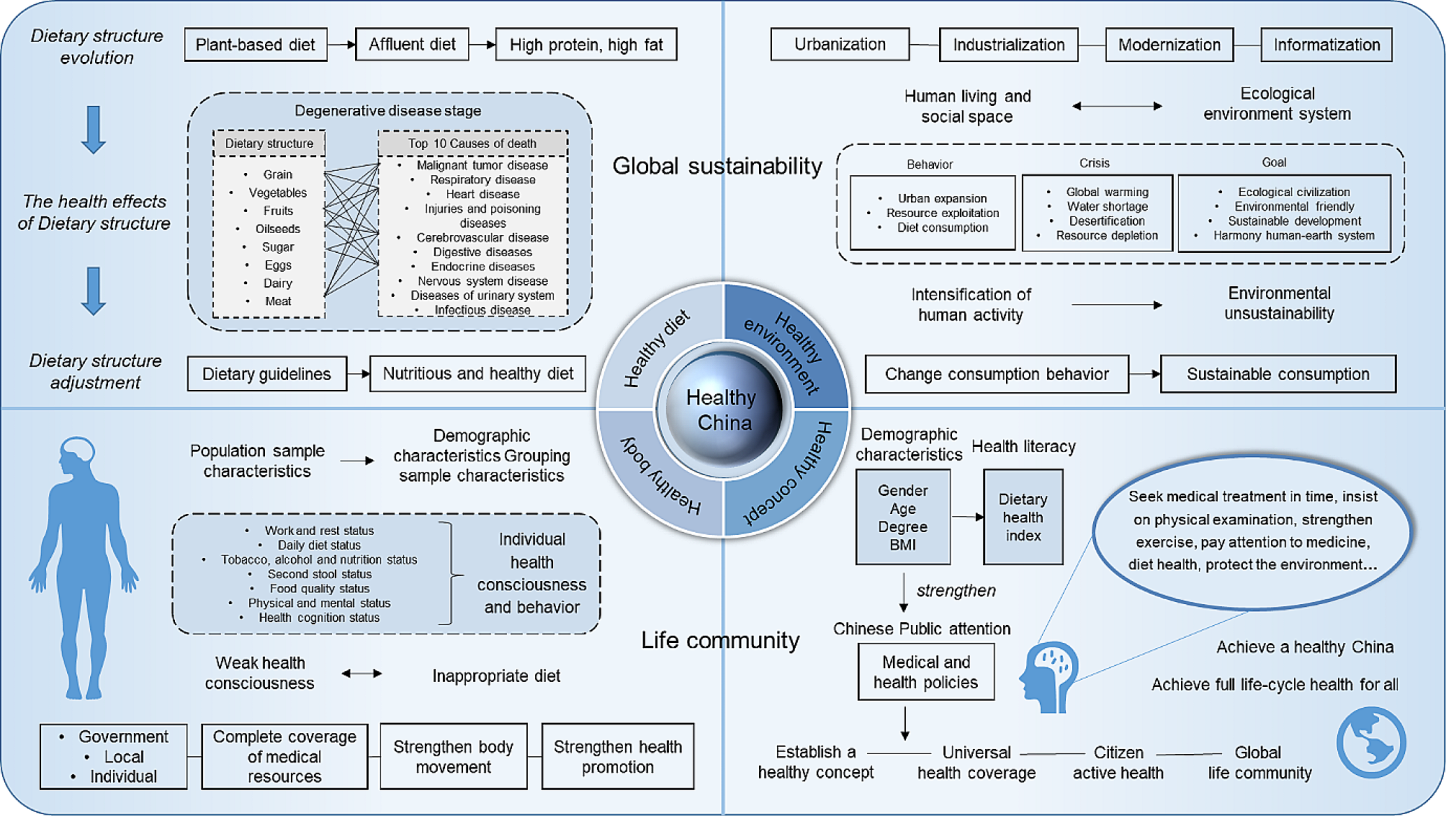
Model diagram of Healthy China path

We believe that the Healthy China strategy is an evolving process, and it should analyze existing issues from a developmental perspective, including their causation. The macro analysis serves to identify prevalent issues in the dietary habits of the Chinese public. Additionally, micro-level individual behavior analysis aims to pinpoint specific health behavior issues among Chinese residents, and to analyze the underlying causes of unhealthy behaviors from the perspective of health awareness. Our findings indicated that the Chinese diet is progressively transitioning towards a stage of degenerative diseases, thereby escalating the risk of chronic and other ailments [41]. Furthermore, the physical health status of the Chinese population remains suboptimal, characterized by inadequate active health awareness and low health literacy rates. The Healthy China Initiative strives to achieve comprehensive health for all citizens, necessitating a shift from reactive treatment to proactive prevention. Therefore, we believe that the future national health strategy should focus on the four health models of “diet health, physical health, conceptual health and environmental health”. It aims to promote the implementation of the Healthy China strategy at both macro and individual levels (Fig. 6).

First, with regards to diet health, it is necessary to pay attention to dietary adjustments and utilize national health policies like the national dietary guidelines. Daily dietary recommendations advocate for the inclusion of whole grains, potatoes, vegetables, fruits, lean meats, poultry, fish, eggs, dairy products, and legumes to fulfill diverse nutritional needs while ensuring a balanced nutrient intake [42]. Furthermore, promoting changes in individual dietary choices through conceptual intervention and transitioning from an animal-based diet to a reasonable and healthy diet are essential.

Second, the health status of individuals significantly influences socioeconomic development, with individuals bearing primary responsibility for their own health. Urgent measures are needed to promote universal health initiatives, residents should be encouraged to proactively engage in preventive measures and enhance physical activity across all domains to uphold optimal health.

Third, individual health consciousness are important factors affecting health level. Therefore, there is a need to foster a culture of proactive health among residents and enhance their health awareness and literacy in order to ensure individual well-being.

Fourth, the combination of the Healthy China strategy and ecological civilization has become instrumental in achieving sustainable development goals. From both macro and individual perspective, promoting the ecological environmental health with the concept of ecological civilization to achieve a sustainable global life community is the real realization of Healthy China. This is not only a great contribution to China, but also a great contribution to the world.

## Conclusion

Diet, consciousness and health affect each other and restrict each other. Diet, as a fundamental human right, is influenced by individual subjective awareness. Moreover, dietary intake will affect individual health. From the perspective of the multi-dimensional correlation of “diet-consciousness-health-life community,” this study deeply analyzed the existing problems of Chinese residents’ health behaviors from both macro and micro perspectives. This study found that the Chinese diet structure has entered the stage of degenerative disease, and individual eating behaviors are greatly affected by demographic characteristics such as gender, age and BMI. The health awareness and concept of Chinese are still neglected in terms of exercise and physical condition. Despite more than a decade of promoting the Healthy China strategy, an effective link between China’s public health system and individuals’ health needs has not been established. This study believes that it should promote the true realization of Healthy China strategy from the perspective of “diet health, physical health, conceptual health and environmental health,” cover all aspects of health care, education, environment and system. The contradiction and restriction between diet, consciousness and health are still the constraints to achieve global public health and the Millennium Sustainable Development Goals of human society. In the future, there is still a need for further exploration towards the reasonable improvement and enhancement of individual health awareness and behavioral concepts.

### Electronic supplementary material

Below is the link to the electronic supplementary material.

Supplementary Material 1

## Data Availability

The data and materials used for the analyses are available by email request to the corresponding author.

## Abbreviation

BMI: Body Mass Index

## References

[1] Zhao W, Yu K, Tan S (2017). Dietary diversity scores: an indicator of micronutrient inadequacy instead of obesity for Chinese children. BMC Public Health.

[2] WHO. 2023. World health statistics 2023: monitoring health for the SDGs, sustainable development goals. https://www.who.int/publications-detail-redirect/9789240074323.

[3] Smyth F (2008). Medical geography: understanding health inequalities. Prog Hum Geogr.

[4] UN. 2022. Human development report 2021-22. https://www.who.int/publications-detail-redirect/9789240074323. Accessed May 3, 2023.

[5] UN, United Nations Millennium Declaration on the United Nations General Assembly. 2000., 8 September 2000. https://www.un.org/zh/documents/treaty/A-RES-55-2.

[6] UN. 2015. The 2030 Agenda for Sustainable Development in September 2015, world leaders adopted at a historic United Nations summit. https://www.un.org/sustainabledevelopment/sustainable-development-goals/.

[7] Grossman M (1972). On the concept of health care and the demand for health. J Polit Econ.

[8] Preston SH, Wang H (2006). Sex mortality differences in the United States: the role of cohort smoking patterns. Demography.

[9] Mukong AK, Van Walbeek C, Ross H (2017). Lifestyle and income-related Inequality in Health in South Africa. Int J Equity Health.

[10] GBD 2019 Pakistan Collaborators (2023). The state of health in Pakistan and its provinces and territories, 1990–2019: a systematic analysis for the global burden of Disease Study 2019. Lancet Glob Health.

[11] Acheson D (1998). Independent inquiry into inequalities in health.

[12] Zatonski W (2007). The East-West Health Gap in Europe–what are the causes?. Eur J Public Health.

[13] Browning CR, Soller B, Jackson AL (2015). Neighborhoods and adolescent health-risk behavior: an ecological network approach. Soc Sci Med.

[14] Lazzeri G, Ciardullo S, Spinelli A (2023). The correlation between adolescent Daily Breakfast Consumption and Socio-Demographic: Trends in 23 European countries participating in the Health Behaviour in School-aged children study (2002–2018). Nutrients.

[15] Du Y, Rong S, Sun Y (2021). Association between frequency of eating away-from-home meals and risk of all-cause and cause-specific mortality. J Acad Nutr Diet.

[16] Kjeldsen EW, Thomassen JQ, Rasmussen KL (2022). Impact of diet on ten-year absolute cardiovascular risk in a prospective cohort of 94 321 individuals: a tool for implementation of healthy diets. Lancet Reg Health Eur.

[17] Neuhouser ML (2019). The importance of healthy dietary patterns in chronic disease prevention. Nutr Res.

[18] Fang YH, Xia J, Lian YY, et al. The burden of cardiovascular disease attributable to dietary risk factors in the provinces of China, 2002–2018: a nationwide population-based study. Lancet Reg Health. 2023;100784. 10.1016/j.lanwpc.2023.100784. 10.1016/j.lanwpc.2023.100784 PMC10485670 37693878

[19] Pant A, Gribbin S, McIntyre D (2023). Primary prevention of cardiovascular disease in women with a Mediterranean diet: systematic review and meta-analysis. Heart.

[20] Wright RS, Waldstein SR, Kuczmarski MF (2017). Diet quality and cognitive function in an urban sample: findings from the healthy aging in neighborhoods of diversity across the Life Span (HANDLS) study. Public Health Nutr.

[21] ONSH. 2020. Healthy People 2030 Framework. https://health.gov/healthypeople/about/healthy-people-2030-framework.

[22] Hasbrouck L (2021). Healthy people 2030: an Improved Framework. Health Educ Behav.

[23] National Health Commission (NHSC). Report on Nutrition and Chronic Diseases of Chinese Residents 2021.

[24] Liu J, Zhang Y (2019). Health status and health disparity in China: a demographic and socioeconomic perspective. China Popul Dev Stud.

[25] Aaby A, Friis K, Christensen B, Rowlands G, Maindal HT (2017). Health literacy is associated with health behaviour and self-reported health: a large population-based study in individuals with cardiovascular disease. Eur J Prev Cardiol.

[26] Šulinskaitė K, Zagurskienė D, Blaževičienė A (2022). Patients’ health literacy and health behaviour assessment in primary health care: evidence from a cross-sectional survey. BMC Prim Care.

[27] GBD 2017 Diet Collaborators (2019). Health effects of dietary risks in 195 countries, 1990–2017: a systematic analysis for the global burden of Disease Study 2017. Lancet.

[28] HLY (2014). Introduction of 2012 Chinese residents health literacy monitoring program. Chin J Health Educ.

[29] Svendsen MT, Bak CK, Sørensen K (2020). Associations of health literacy with socioeconomic position, health risk behavior, and health status: a large national population-based survey among Danish adults. BMC Public Health.

[30] Sørensen K, Van Den Broucke S, Fullam J, Doyle G, Pelikan J (2012). Health literacy and public health: a systematic review and integration of definitions and models. BMC Public Health.

[31] Clark MA, Springmann M, Hill J, Tilman D (2019). Multiple health and environmental impacts of foods. Proc Natl Acad Sci U S A.

[32] Chinese Nutrition Society(CNS). 2021. Dietary Guidelines for Chinese Residents Scientific Research Report 2021.

[33] He Y, Li Y, Yang X (2019). The dietary transition and its association with cardiometabolic mortality among Chinese adults, 1982–2012: a cross-sectional population-based study. Lancet Diabetes Endocrinol.

[34] Wang L, Zhou B, Zhao Z (2021). Body-mass index and obesity in urban and rural China: findings from consecutive nationally representative surveys during 2004-18. Lancet.

[35] Pan XF, Wang L, Pan A (2021). Epidemiology and determinants of obesity in China. Lancet Diabetes Endocrinol.

[36] Zhu YY, Wang ZW, Zhu XH (2023). New reflections on food security and land use strategies based on the evolution of Chinese dietary patterns. Land use Policy.

[37] Zhu XH, Zhang Y, Zhu YY, Li YR (2023). The shift to plant-based dietary patterns in China would have significant effects on cultivated land resources. Sci Bull.

[38] Springmann M, Wiebe K, Mason-D’Croz D (2018). Health and nutritional aspects of sustainable diet strategies and their association with environmental impacts: a global modelling analysis with country-level detail. Lancet.

[39] Willett W, Rockström J, Loken B (2019). Food in the Anthropocene: the EAT-Lancet Commission on healthy diets from sustainable food systems. Lancet.

[40] Zhu YY, Zhang Y, Zhu XH (2023). The evolution process, characteristics and adjustment of Chinese dietary guidelines: a global perspective. Resour Conserv Recycl.

[41] Popkin BM (2004). The nutrition transition: an overview of world patterns of change. Nutr Rev.

[42] Chinese Nutrition Society(CNS) (2022). Chinese Dietary guidelines 2022.

[43] National Health Commission of the People’s Republic of China. Measures for Ethical Review of Human Life Science and Medical Research. (2023, Chapter III, Article 32). https://www.gov.cn/zhengce/zhengceku/2023-02/28/content_5743658.htm.

