# Supplementary material for: Realization path and connotation of the Healthy China strategy: macroscopic perspective of dietary structure and the entry of individual health consciousness
Source: BMC Public Health. 2024 Apr 23;24:1120. doi: 10.1186/s12889-024-18557-z (PMC11040960; doi:10.1186/s12889-024-18557-z)
Supplement: Supplementary file 1 — Supplementary Material 1 [file 12889_2024_18557_MOESM1_ESM.docx]

Supplementary Information for

**Realization path and connotation of the Healthy China strategy: Macroscopic perspective of dietary structure and the entry of individual health consciousness**

**Table of Contents**

Tables. S1 to S3

**Supplementary TableS1 to S2**

**Table S1.**

Main survey contents of individual health awareness and behavior questionnaire.

| **Survey contents** | **Survey questions** | **Survey indicators and sub-items** | | | | |
| --- | --- | --- | --- | --- | --- | --- |
|  |  | **1** | **2** | **3** | **4** | **5** |
| **Work and rest** | **About getting up every morning** | After 9:00 | Before 9:00 | Before 8:00 | Before 7:00 | Before 6:00 |
|  | **About going to bed every night** | Before three or four in the morning | Before one or two in the morning | Every night before 12:00 | Every night before 11:00 | Every night before 10:00 |
|  | **Bedtime routine** | You must check your phone before going to bed | Check your phone often before going to bed | Occasionally check your phone before bed | Don't check your phone before bed | You must check your phone before going to bed |
|  | **About staying up late** | Stay up late almost every day | Often stay up late | Occasionally stay up late | Don't stay up late | Stay up late almost every day |
|  | **About lunch break** | Sometimes take a lunch break | Have the habit of taking a lunch break all year round | No habit of taking lunch breaks | Sometimes take a lunch break | Have the habit of taking a lunch break all year round |
|  | **About Insomnia** | Insomnia almost every day | Often have insomnia | Occasional insomnia | No insomnia | Insomnia almost every day |
|  | **About how much sleep you get each day** | Less than 4 hours | About four to six hours | About 6 to 8 hours | About eight hours or more |  |
|  | **About sleep quality** | Very bad | Poor | Normal | Right | Fine |
| **Daily diet** | **About feeling full every time you eat** | Eat until you feel full; Eat until you're 20 or 30 percent full | Eat until you feel full; Eat half full | Eat until you feel half full |  |  |
|  | **About the harm of "three white" (refined rice flour white sugar)** | Incomprehension | Know something about | Better understand |  |  |
|  | **About the harm of gutter oil** | Incomprehension | Know something about | Better understand |  |  |
|  | **About eating out** | Eat out often | Eat out occasionally | Eat mostly at home |  |  |
|  | **About eating meat and vegetables** | Meat and vegetable based | Meat and vegetables are equally divided | Vegetable-based | Eating mostly vegetables |  |
|  | **About fruit** | Without eating | Occasionally eat | Eat often | Almost every day |  |
|  | **About brown rice** | Never heard of | I've heard of it, but I've never eaten it | Heard about it and ate it |  |  |
|  | **About whole grains** | Without eating | Occasionally eat | Eat often |  |  |
|  | **On dietary regularity** | Often irregular | Occasionally and irregularly | Regular |  |  |
|  | **About breakfast** | Skip breakfast | Do not eat often | Sometimes I don't eat | Almost every day |  |
|  | **About the amount of dinner** | Eat well; Skip dinner | Eat more fully; Have a bite of | Eat until half full |  |  |
|  | **About the size of the dinner (breakfast, lunch)** | The biggest dinner | Dinner is big | So-so dinner | Dinner is simple. |  |
|  | **About dinner time** | Skip dinner | After 8:00 p.m | Before 8:00 p.m | Before 7:00 p.m | Before 6:00 p.m |
|  | **About late night snacks** | Eat late night snacks almost every day | Eat late night snacks often | Have an occasional late night snack | No late night snacks |  |
|  | **About hunger** | I often feel hungry | Occasionally I feel hungry | No hunger |  |  |
|  | **About eating habits** | Eat when you're hungry | Eat when you arrive |  |  |  |
|  | **About food taste** | Very strong taste | Strong taste | Occasionally heavy taste | Light taste |  |
|  | **About drinking water** | Drink only colored or carbonated beverages, no water | Often replace water with colored or carbonated beverages | Drink less water every day | The amount of water you drink every day feels OK | The amount of water you drink every day feels adequate |
|  | **About pastries, instant noodles and other processed packaged food** | Often buy to eat | Buy and eat occasionally | Barely eat |  |  |
|  | **About eating buffet** | I always eat until I'm full | Just eat until you're full | Eat freely at a time |  |  |
|  | **About colored or carbonated drinks** | Drink often | Drink occasionally | Barely drink |  |  |
| **Tobacco, alcohol and nutrition** | **About smoking** | Regular smoker | Occasional smoker | No smoking |  |  |
|  | **About drinking (mainly liquor)** | Drink a lot | Drink occasionally | Keep one's nose clean |  |  |
|  | **About taking health products^1^** | Eat health products regularly | Eat health products once in a while | Don't eat health products |  |  |
| **Bowel conditions** | **About defecation** | You only have one bowel movement every day | Defecate every four or five days | Defecate once every two or three days | Although I defecate every day, I find it difficult | Smooth bowel movements every day |
|  | **About my own urine and feces** | Never observed | Occasionally observed | Frequent observation | Stay up late at night |  |
|  | **Do you think defecation is a sign of good health** | Incomprehension | Maybe | It should be | Have never had |  |
|  | **About nocturia** | Get up more than 4 times at night | Get up 3-4 times at night | Get up twice at night |  |  |
|  | **About hemorrhoids** | Had been suffering from hemorrhoids | I had hemorrhoids, but I'm fine | Occasionally I get hemorrhoids |  |  |
| **Food quality situation** | **About food Additives** | incomprehension | Know a little about its dangers | Know more about its dangers |  |  |
|  | **About food quality** | Great trust | Comparative trust | General trust | Distrust |  |
| **Motion condition**  **Physical and mental condition** | **About sitting** | Basically sedentary all day | Be sedentary | Occasionally sedentary | Sit down soon |  |
|  | **About sun exposure** | Rarely go out in the sun | Get out in the sun |  |  |  |
|  | **About sports** | Basic inactivity | Occasional exercise | Exercise regularly | Basic daily exercise |  |
|  | **About exercise time (if any)** | Within 1 hour a day | 1-2 hours a day | About 3 hours a day | More than 3 hours a day |  |
|  | **On weight loss** | A little fat, but not losing weight | Lost weight but didn't succeed | Lost weight and succeeded | Eunomia |  |
|  | **About life stress** | Life is very stressful | Feeling the pressure of life is greater | Life is stressful | No stress in life |  |
|  | **About anxiety** | Feel anxious | Feel more anxious | Feeling a little anxious | There's no sense of anxiety |  |
|  | **About depression** | Feel depressed | Feel more depressed | Feeling a little depressed | I don't feel depressed |  |
|  | **About tinnitus** | Persistent tinnitus | Constant tinnitus | Occasional tinnitus | Never had tinnitus |  |
|  | **About working hours each day** | About 12 hours or more | About 12 to 10 hours | About 10 to 8 hours | Less than 8 hours |  |
|  | **About work intensity** | It feels like a lot of work | Feel the work intensity is greater | I feel the work intensity is average | It feels easier to work |  |
|  | **About fatigue** | Chronic sensory fatigue | Often feel tired | Occasionally feel tired | Don't feel tired |  |
|  | **About whether you feel tired easily** | I feel tired easily | I feel tired more easily | I feel tired sometimes | I don't feel tired easily |  |
| **Health perception** | **About nutritional status** | The more nutrition, the healthier | incomprehension | Excess nutrition can damage health |  |  |
|  | **About chronic diseases Whether to take medicine for life** | Incomprehension | Don't take medicine all your life | Take medicine for life |  |  |
|  | **Have you ever reflected on why you got sick** | Incomprehension | Have not reflected | Have thought over | Better understand |  |
|  | **About the body's ability to heal itself** | Incomprehension | Never heard of | Know of | Better understand |  |
|  | **About the attitude toward surgical success** | A successful operation is a cure; incomprehension | A successful operation is a partial cure | A successful operation and a cure are two different things |  |  |
|  | **About illness** | Don't tell the doctor | Selectively tell the doctor in part | Tell the doctor everything when you see him |  |  |
|  | **If you get sick, you will see a doctor in time** | Avoid medical treatment, do not go to the doctor | If you do not seek medical attention in time, you will suffer from serious illness and be dragged off by minor illness | Seek medical attention promptly | The hospital is the auxiliary, oneself is the main |  |
|  | **Choose Western medicine or Chinese medicine** | Look only at "miracle doctors" | Only Western medicine; Look only at traditional Chinese medicine | Integration of traditional Chinese and western medicine |  |  |
|  | **About Rehabilitation Medicine^2^, rehabilitation treatment ^3^** | Have no idea | Know a little | General understanding |  |  |
|  | **Responsibility for health** | In hospital; incomprehension | Rely on yourself | Hospital is the main thing, oneself is the aid |  |  |
|  | **About physical examination** | Never had a physical. | Check up every few years (more than 3 years) | Check up every few years  (two or three years) | Have an annual physical examination | Physical examination twice a year or more |
|  | **About trust in doctors and hospitals** | Distrust | Comparative trust | More trust | Complete trust |  |
|  | **About adverse drug reactions** | Incomprehension | Know a little | General understanding | Better understand |  |
|  | **About misdiagnosis rate** | Incomprehension | Know a little | General understanding | Better understand |  |
|  | **About over medicalization** | Incomprehension | Know a little | General understanding | Better understand |  |
|  | **About indicators Normal equals healthy** | Incomprehension | Normal indicators are good health |  |  |  |
|  | **Do you feel healthy?** | Very unhealthy | Unhealthy. | Sub-health^4^ | Relatively healthy | Very healthy |

Notes:

**1**. Health products claims to have specific health functions, or can supplement vitamins, minerals and other nutrients, suitable for specific people to eat, with the regulation of body function, not for the purpose of treating disease food.

**2**. Rehabilitation Medicine Rehabilitation medicine is an emerging discipline, which is a new concept that appears in the middle of the 20th century. Rehabilitation medicine and preventive medicine, health medicine, clinical medicine and known as the "four major medicine", it is a medical discipline to eliminate and reduce people's dysfunction, make up for and rebuild people's lack of function, and try to improve and improve all aspects of human function, that is, the prevention of dysfunction, diagnosis, evaluation, treatment, training and treatment of medical disciplines.

**3**. Rehabilitation therapy refers to the treatment of physical and mental dysfunction or disability caused by injury, disease, developmental defects and other factors to return to normal or close to normal. It is an important part of rehabilitation medicine. Including physical therapy, movement therapy, occupational therapy, speech therapy, swallowing therapy, psychotherapy, stylistic therapy, rehabilitation engineering (prosthetics and orthotics) and traditional Chinese medicine (traditional Chinese medicine, acupuncture, gua sha, cupping, massage, etc.).

**4.** A state of being between health and disease. People in sub-health cannot reach the standard of health, which is manifested as the symptoms of reduced vitality, function and adaptability in a certain period of time, but does not meet the clinical or sub-clinical diagnostic standards of modern medicine related diseases. The main characteristics of sub-health include: ① all kinds of symptoms reflected by the feeling of physical and mental inadaptability, such as fatigue, weakness, mood change, etc., the status of which is difficult to identify in a considerable period of time; ② various weakness caused by age-inappropriate tissue structure or physiological function decline; ③ the state of microecological imbalance; ④ Prepathologic changes of some diseases.

**Table S2.**

Results of binary Probit regression analysis.

| Variables | Regression coefficient | Standard error | *z* | *p* | 95% CI |
| --- | --- | --- | --- | --- | --- |
| Gender | 0.232 | 0.067 | 3.451 | 0.001 | 0.100 ~ 0.363 |
| Degree | 0.008 | 0.031 | 0.263 | 0.793 | -0.053 ~ 0.069 |
| Age | 0.366 | 0.027 | 13.37 | 0 | 0.313 ~ 0.420 |
| BMI | -0.111 | 0.049 | -2.279 | 0.023 | -0.207 ~ -0.016 |
| Intercept | -0.767 | 0.189 | -4.068 | 0 | -1.136 ~ -0.397 |

**Table S3.**

Results of binary Logit regression analysis.

| Variables | Regression coefficient | Standard error | *z* | *p* | OR | OR(95% CI) |
| --- | --- | --- | --- | --- | --- | --- |
| Gender | 0.375 | 0.110 | 3.405 | 0.001 | 1.455 | 1.173 ~ 1.806 |
| Degree | 0.017 | 0.051 | 0.333 | 0.739 | 1.017 | 0.920 ~ 1.124 |
| Age | 0.600 | 0.047 | 12.878 | 0.000 | 1.822 | 1.663 ~ 1.996 |
| BMI | -0.190 | 0.081 | -2.354 | 0.019 | 0.827 | 0.706 ~ 0.969 |
| Intercept | -1.442 | 0.267 | -5.405 | 0.000 | 0.236 | 0.140 ~ 0.399 |
